# Temporal profiling of haptoglobin and cytokines in feedlot cattle with bovine respiratory disease

**DOI:** 10.3389/fvets.2025.1713337

**Published:** 2026-01-07

**Authors:** Carol G. Chitko-McKown, Bailey N. Engle, Gary L. Bennett, Larry A. Kuehn, Keith D. DeDonder, Michael D. Apley, Gregory P. Harhay, Michael L. Clawson, Brad J. White, Robert L. Larson, Sarah F. Capik, Brian V. Lubbers

**Affiliations:** 1 USDA-ARS US Meat Animal Research Center, Clay Center, NE, United States; 2 College of Veterinary Medicine, Kansas State University, Manhattan, KS, United States

**Keywords:** bovine respiratory disease, cattle, cytokines, haptoglobin, interferon-γ, interleukin-1β, interleukin-6, tumor necrosis factor-*α*

## Abstract

Inflammatory cytokines and haptoglobin (HPT) were measured from 28 head of cattle that presented with bovine respiratory disease after being purchased and shipped to a common feedlot and assigned to either control or metaphylaxis treatment groups. Blood samples were obtained on the day of purchase (D0) and again at the time of diagnosis (S0) and 5 days after treatment (S5). Plasma was harvested and stored at −80 °C until assayed for the cytokines IFN-γ, IL-1β, IL-6, TNF-α, and HPT. Our objectives in this study were to determine differences between baseline (D0), day of diagnosis (S0) and post-treatment (S5), as well as to identify correlations among cytokine and HPT concentrations over time and by treatment. Interferon-gamma and IL-1β concentrations did not differ between D0 and S0 or S5. Tumor necrosis factor-*α* concentrations differed between D0 and both S0 and S5; however, no differences were observed between S0 and S5. Interleukin-6 and HPT concentrations differed between D0 and S0, D0 and S5, and between S0 and S5. Neither control nor metaphylactic treatment significantly impacted cytokine or HPT concentrations, nor did the state of origin. The strongest significant correlations among cytokine-timepoint combinations were observed for IL-1β and TNF-α S0 (0.93), and S5 (0.91). Both IL-6 and HPT increased from baseline to the time of BRD diagnosis and treatment, and HPT exhibited the most rapid decline after treatment (S5), suggesting that it may be the most useful single measurement for indicating both sickness and resolution post-treatment.

## Introduction

Bovine respiratory disease (BRD) is a multifactorial illness involving viruses, bacteria, stress, and environmental and shipping-related factors (1). It represents an economic burden on the cattle industry and a major animal welfare issue, with costs associated with reduced efficiency and the use of antibiotics required to control infection (2). Differences in animal production/management routines add to the variables involved, with animals being auctioned in sale barns on different planes of nutrition, as well as receiving different preventative vaccinations or anthelminthic treatment (3). When animals under stress from shipping are mixed, and respiratory pathogens are shared, many of the signals of BRD are signs of inflammation (4). When cells of the immune system come into contact with pathogens, for example, lipopolysaccharides from the outer membrane of a bacterium such as *Mannheimia haemolytica*, a cascade of cytokines and acute-phase proteins is released that leads to fever, malaise, and cachexia (5). Haptoglobin (HPT) is an acute phase protein that is produced in the liver upon stimulation by inflammatory cytokines, including interleukin-1β (IL-1β), interleukin-6 (IL-6), and tumor necrosis factor-*α* (TNF-α) (6). The ability to measure these metabolites early in infection may allow for earlier treatment and earlier resolution of infection, as well as diagnostic indicators of BRD infections.

We have previously shown the differences in the profiles of the inflammatory cytokines IL-1β, IL-6, TNF-α, and interferon-γ (IFN-γ), and the acute phase protein HPT in animals upon purchase at a sale barn (D0), arrival and processing at a feedyard (D1), and on the 9th (D9) and 28th days (D28) in the feedyard (7). When comparing levels of cytokines and HPT between sick and control animals, we found that the metabolite that was most associated with the onset of BRD was HPT measured on D9. Our objectives in this study were to determine if inflammatory cytokine and HPT concentrations in peripheral blood collected when cattle were identified as having BRD prior to treatment (S0) were different from baseline concentrations collected on D0 at purchase, as well as at 5 days post-treatment (S5), and if there were significant correlations among the cytokine and HPT concentration–time combinations.

## Materials and methods

All activities were reviewed and approved by the Kansas State University Institutional Animal Care and Use Committee (#3338).

### Animals

Twenty-eight animals out of a previously described population consisting of 180 head of cattle, considered to be at high risk for BRD, were diagnosed with it during a 28-day trial (7–9). These animals were purchased at sale barns in three different states (Kentucky, Missouri, and Tennessee), shipped to a common feedlot (approximately 726, 172, and 841 miles, respectively), and assigned to a control (CO) or gamithromycin metaphylactic treatment (MET). Upon processing at the feedlot, animals were examined to ensure that no clinical signs of BRD were present at intake, and in addition to their assigned treatments, received a modified live viral respiratory vaccine, a clostridial vaccine, an anthelminthic, and a growth implant. A veterinarian masked to treatment assignment observed the animals daily for signs of BRD. The diagnosis of BRD was based on cattle having a rectal temperature of ≥ 40.0 °C (≥104.0 °F) and a clinical score of ≥1, indicating signs of weakness and depression as previously described (8, 9). Out of the entire 180-head population, only 28 were included in this analysis and were diagnosed with BRD. These animals were sampled pre-treatment (S0), then administered gamithromycin at the labeled dose, and were sampled again 5 days after diagnosis [S5; (7–9)].

### Blood sampling

Whole blood was collected by jugular venipuncture into 10 mL Vacutainer tubes containing ethylenediaminetetraacetic acid as an anticoagulant (BD, Franklin Lakes, NJ; #366643) from cattle at the sale barn of purchase and was used as the baseline sample (D0), at the time of diagnosis of BRD (S0), and 5 days after treatment for BRD (S5). Blood samples were kept on wet ice and were transported to the US Meat Animal Research Center in Clay Center, NE, within 24 h. Whole blood was centrifuged at 1,200 × *g* for 20 min to isolate plasma, which was aliquoted into cryovials and stored at −80 °C until assayed (7).

### Metabolite analysis

The bovine inflammatory cytokines IL-1β, TNF-α, IL-6, and IFN-γ were analyzed in duplicate using the Mesoscale Diagnostics (MSD, Rockville, MD) assay platform as previously described (7). The plates were read on a MSD QuickPlex SQ120 imager, and data were analyzed using MSD Workbench 4.0 software (MSD). Standards were run in duplicate on each plate with ranges of 0–50,000 pg/ml for IL-1β and TNF-α, 0–10,000 pg./mL IL-6, and 0–5,000 pg/ml for IFN-γ (7, 10). All samples were analyzed undiluted and were diluted as required up to 1:10 to obtain readings within the ranges of the standards.

Bovine HPT was measured using a commercially available sandwich ELISA (Immunology Consultants Laboratory, Inc., Portland, OR, #E-10HPT), as per the manufacturer’s instructions, as previously described (7), with a range of 15.6–1,000 ng/mL. Concentrations were determined using four-parameter logistic curve fitting^1^ as previously described. Standards were run in duplicate on each plate with a range of 0–1,000 ng/mL (7). All samples were analyzed undiluted and were diluted as required up to 1:2000 to obtain readings within the range of the standards.

### Statistical analysis

Only animals that were diagnosed and received treatment were included in the analysis (*n* = 28). Technical duplicates of each assay were averaged for each animal by sampling day. A natural log transformation was applied to all cytokine data prior to analysis (7); however, HPT did not exhibit a skewed distribution and was therefore left untransformed. Assay concentrations on D0, S1, and 5 days after treatment (S5) were evaluated using the PROC MIXED procedure in SAS. Fixed effects included sampling day, state of origin, treatment group, and assay plate number for all analyses, except for HPT, which did not include the effect of assay plate. Fixed effects were tested on the animal, except for day and day interactions, which were tested on the residual. Unadjusted pairwise differences between least squares means for cytokines were estimated on the natural log scale and back-transformed, and differences in HPT were estimated on the observed scale. Back transformations were a direct exponentiation of model estimates. Standard errors of least squares means were used to calculate 95% confidence intervals prior to back-transformation. Pearson’s correlations between natural log-transformed cytokine concentrations and the untransformed HPT by day were calculated and plotted using *corrplot* in R (versions 0.95 and 4.5.1, respectively).

## Results and discussion

Two animals were identified as sick on D4, two on D6, one on D7, one on D9, two on D10, one on D11, six on D13, three on D14, two on D15, two on D16, four on D18, and two on D19 (mean day of BRD diagnosis = 12.8). None were diagnosed on the sampling days identified in our previous study (7). Nineteen animals were in the CO group, and nine were in the MET group. Ten originated in Kentucky, nine originated in Tennessee, and nine originated in Missouri. The samples taken from the 28 animals with BRD were evenly distributed across seven assay plates (four per plate; 7). Despite the animals being considered at high risk for BRD, the total morbidity throughout the length of the study was less than expected (8). There was no mortality from BRD in the herd of 180 animals. Of the 19 control animals with BRD, 2 suffered from treatment failure, and of the 9 mass-medicated animals with BRD, 2 suffered from treatment failure (8). Two of the four treatment failure animals harbored *M. haemolytica* strains containing macrolide resistance genes (9).

In our previous study, cases were retroactively matched with control samples on D0, D1, D9, and D28. However, when animals were diagnosed with BRD and then treated (S0), no corresponding samples were collected from control animals because it was unknown which of the 180 animals would present with BRD during the 28-day study period. Therefore, samples obtained at S0 and S5 needed to be analyzed separately from the other time points using a different statistical approach. When data were analyzed by treatment group, state of origin, or plate, no significant differences were found for any of the analytes measured, and no interaction with day was found to be significant (results not shown).

Mean concentrations of IL-1β and IFN-γ did not change from D0 to S0 nor S5; however, TNF-α concentrations were significantly different between D0 and S0 as well as between D0 and S5. IL-6 concentrations differed across all three sampling times, increasing approximately five times between D0 and S0, then decreasing by half between S0 and S5. Similarly, HPT concentrations increased more than 40 times between D0 and S0, then decreased by approximately half on S5 (Table 1).

**Table 1:** Differences in least squares means^1^ by day in plasma samples from 28 high-risk beef cattle identified as clinical bovine respiratory disease (BRD) cases.

Day^2^^,^^3^	D0	95% CI	S0	95% CI	S5	95% CI
IL-1β	4,104	1,421–11,850	7,693	2,686–22,031	9,166	3,201–26,250
TNF-α	4,307^a^	1809–10,250	8,940^b^	3,779–21,149	9,237^b^	3,905–21,852
IL-6	20.8 ^a^	12.1–27.4	101.6^b^	60.2–132.6	54.5^c^	32.1–71.4
IFN-γ	11.2	8.7–14.3	12.8	10.0–16.3	10.3	8.0–13.1
HPT	79,940^a^	−1,095,497–1,255,377	3,469,559^b^	2,313,022–4,626,096	1,996,486^c^	839,949–3,153,023

^a,b,c^Significant unadjusted pairwise differences of at least a *p*-value of < 0.05, estimated on the natural log transformed scale.

1 Least squares means were estimated on a natural log scale and back-transformed for publication. Estimated standard errors were used to calculate confidence intervals prior to back transformation.

2 Baseline sample (D0), sample at the time of diagnosis of BRD (S0), and 5 days after treatment for BRD (S5), interleukin-1β (IL-1β), interleukin-6 (IL-6), tumor necrosis factor-α (TNF-α), interferon-γ (IFN-γ), and haptoglobin (HPT).

3 Cytokines were all measured in pg/mL, and HPT was measured in ng/mL.

**Figure 1:**
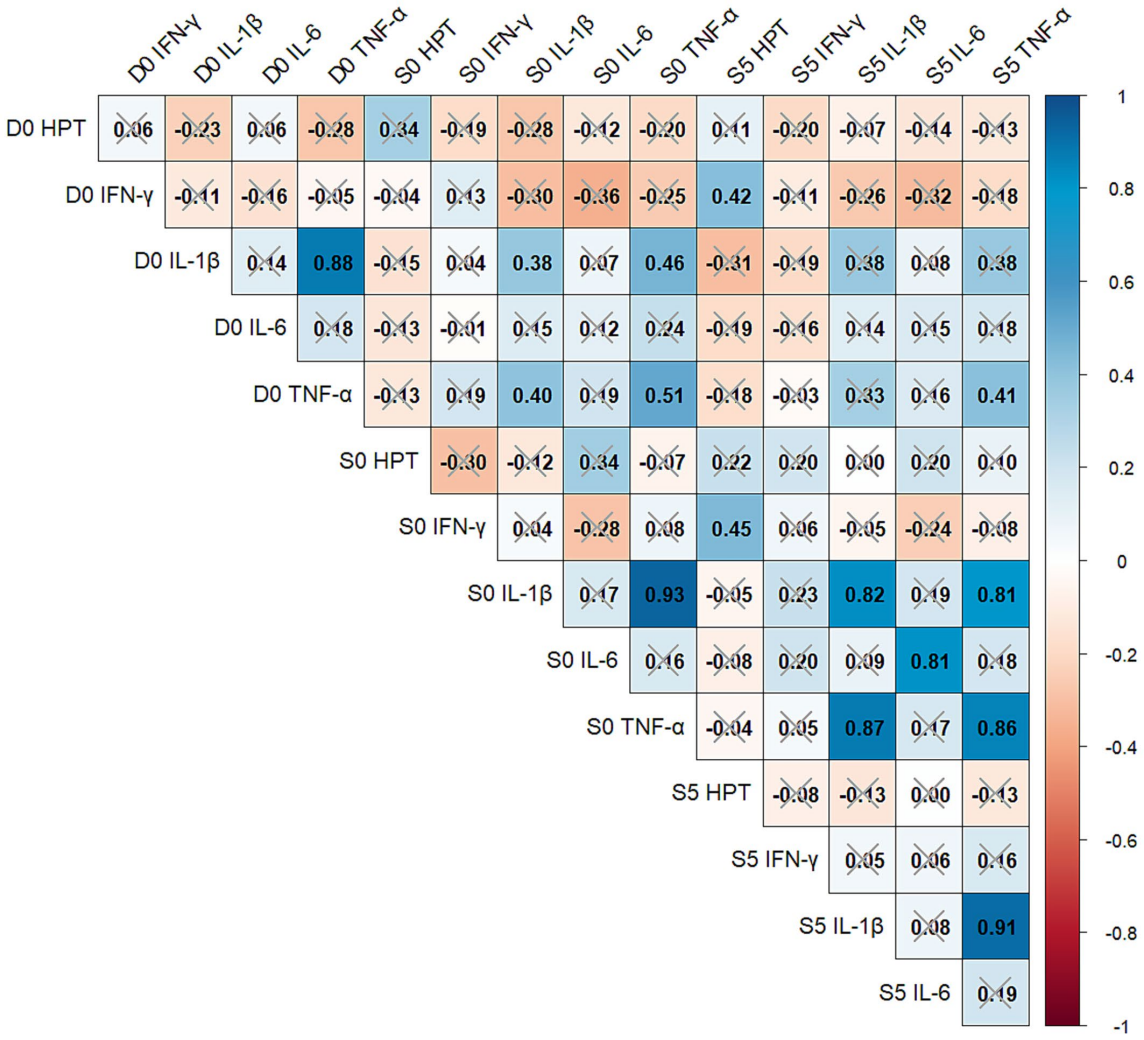
Pearson’s correlations between haptoglobin and natural log-transformed cytokine levels from 28 high-risk beef cattle diagnosed with clinical bovine respiratory disease (BRD) at baseline sample (D0), at the time of diagnosis of BRD (S0), and 5 days after treatment for BRD (S5). Associations between assay-timepoint combinations were deemed to be significantly different from zero if *p* < 0.05, with non-significant correlations noted by a grey “x.” IL-1β,interleukin-1β; IL-6, interleukin-6; TNF-α, tumor necrosis factor-α; IFN-γ, interferon-γ; HPT,haptoglobin.

HPT concentration was significantly correlated with IFN-γ concentration (S5 HPT with D0 and S0 IFN-γ; 0.45 and 0.43, respectively; *p* < 0.03) and was not correlated with any other measured cytokines at any time point. Interferon-γ concentrations were also not significantly associated with any other cytokines. Interleukin-6 concentrations were significantly correlated between S0 and S5 at 0.81 (*p* < 0.0001). Interestingly, IL-1β and TNF-α concentrations were significantly (*p* < 0.0001) correlated at D0 (0.88), S0 (0.93), and S5 (0.91). Interleukin-1β concentrations on D0 were significantly correlated with S0 (0.38, *p* = 0.05) but not with S5 (0.38, *p* = 0.06); however, S0 and S5 were significantly correlated (0.82, *p* < 0.0001). Significant TNF-*α* correlations between D0 and S0 (0.51, *p* = 0.006) and D0 and S5 (0.41, *p* = 0.03) were identified (Figure 1).

Green et al. (11) compared cattle that moved through a commercial auction to cattle that were directly shipped to a feedlot while presenting with BRD. Surprisingly, they found that those who moved through an auction had increased gene expression related to innate immunity and viral defense. Counter to conventional wisdom that assumes that the more cattle are mixed, the more BRD may occur, their results may explain why only 28 of 180 head specifically purchased from multiple sale barns in our study were diagnosed with BRD. Additionally, cattle in this study were assigned to pens based on the location of purchase, so mixing occurred on the feedlot but not at the pen level (8, 9).

In a study of individually housed veal calves, Berman et al. (12) used thoracic ultrasonography (TUS) and HPT concentrations to develop a scoring system for identifying BRD. Calves were diagnosed with active BRD if their HPT concentration was ≥0.25 g/L. The mean HPT concentration in our animals on S0 was 3.470 g/L (3,469,559 ng/mL). In a study evaluating the accuracy of different field techniques, including HPT, to diagnose BRD in dairy calves, Decaris et al. (13) found that although all techniques tested had limitations for the exclusive identification of BRD, the measurement of HPT concentration was highly sensitive. Burdick Sanchez et al. (14) found that steers produced an earlier acute phase protein response to a viral and bacterial challenge than heifers, and that there was an overall difference between their innate immune responses; however, the differences between the sexes were not significant for HPT. The cattle in our study were bulls and steers (8).

In a study of naturally occurring BRD in bulls on feedlots, Roualt et al. (15) clinically assessed the animals on D5, D14, D21, and D28 to identify potential cases. Controls were retroactively selected. Plasma cytokine levels were measured with a bead-based multiplex assay including IL-1β, IL-6, TNF-α, and IFN-γ. In their study, there were no differences between the sick and the control animals for any of the cytokines measured; however, because the animals were not observed every day, the actual cytokine concentrations at the onset of sickness may have been missed. However, IFN-γ levels were positively associated with disease severity and treatment relapse. They conclude that although not diagnostic, cytokine levels may prove to have prognostic potential (15). In a trial aimed at determining the diagnostic and prognostic effect of cytokines and acute phase proteins, El-Deeb et al. (16) detected higher serum levels of IL-1β, TNF-α, IFN-γ, and HPT in feedlot calves with BRD compared to control calves. Akter et al. (5) measured cytokines and HPT in newly received beef calves in a commercial stocker farm and found that IL-1β, IL-8, and TNF-α were variable; however, calves that received multiple treatments for ongoing BRD had the highest HPT concentrations and were significantly different based on treatment status and lung consolidation, demonstrating that HPT can potentially be used as a biomarker for BRD. Lowie et al. (17) explored the association between lung consolidation and acute-phase protein levels in cattle with BRD. They found that lung consolidation was associated with greater levels of HPT (17). Saher et al. (18) found that levels of HPT were significantly increased in *Mycoplasmopsis bovis* (formerly *Mycoplasma bovis*) infected calves compared to healthy controls. Even though HPT decreased in this study from S0 to S5, the level was still significantly higher at S5 than at D0, indicating that HPT may be a good marker to detect that animals are recovering from BRD, although pathogens may not have been completely cleared after initial treatment of symptoms.

In conclusion, the single metabolite most indicative of both the onset of BRD and treatment success in this study was HPT. Although HPT is non-specifically induced, it is more quickly cleared from circulation, which may result in it being a good candidate for diagnosis and prognosis of a multi-factorial disease such as BRD. If the four animals with treatment failure were removed from our analysis, the levels of HPT at S5 may have been lower than those we obtained by including all 28 animals. Our findings indicate haptoglobin may be a BRD diagnostic aid as values increased at the time of treatment compared to arrival or post-treatment levels. Additional studies should measure HPT concentrations in cattle from arrival through diagnosis, treatment, and either successful recovery or morbidity. Only with additional data can HPT concentration thresholds associated with successful recovery be determined.

## Data Availability

The raw data supporting the conclusions of this article will be made available by the authors, without undue reservation.

## Footnotes

1
http://ELISAnalysis.com
